# Supplementary material for: Complexome profiling of the Chlamydomonas psb28 mutant reveals TEF5 as an early PSII assembly factor
Source: Plant Cell. 2025 Mar 17;37(6):koaf055. doi: 10.1093/plcell/koaf055 (PMC12164590; doi:10.1093/plcell/koaf055)
Supplement: koaf055_Supplementary_Data [file koaf055_supplementary_data.zip › psb28_interactive.zip!/plots/intensity/qualityPlots/peptides/LHCA4_Cre10.g452050.t1.2.html]

### Cre10.g452050.t1.2

Trivial name: LHCA4  
SeqCoverage [median]: 48.10  
Mass: 28.70 kDa  
  
 

| Sequence | MissedCleavage | Mass [Da] | wtIntensity | muIntensity | Score |
| --- | --- | --- | --- | --- | --- |
| ASAVPENVK | 0 | 913.49 | 33907.58 | 15706.61 | 140.09 |
| ASAVPENVKEAR | 1 | 1269.67 | 31292.29 | 16505.50 | 117.86 |
| EIKNGRLAMLAFAGFIGQAYTTGTTPLK | 2 | 2968.58 | 2951.18 | 0.00 | 51.59 |
| EWIDAWK | 0 | 946.45 | 120316.05 | 28752.22 | 128.75 |
| LAMLAFAGFIGQAYTTGTTPLK | 0 | 2271.20 | 262183.13 | 38925.40 | 178.72 |
| LKWYAQAELMNAR | 1 | 1592.81 | 57822.67 | 6138.93 | 169.98 |
| LPDGNEPGYPGGIFDPFGWSK | 0 | 2249.04 | 106525.29 | 0.00 | 153.97 |
| LPDGNEPGYPGGIFDPFGWSKGDIK | 1 | 2662.27 | 8253.94 | 0.00 | 86.01 |
| NGRLAMLAFAGFIGQAYTTGTTPLK | 1 | 2598.36 | 3780.63 | 0.00 | 103.67 |
| NLSTHLADPWSTTVWQNDLAR | 0 | 2424.18 | 92019.57 | 0.00 | 184.60 |
| PGSANQDPIFTNNK | 0 | 1501.72 | 16961.77 | 5098.77 | 240.65 |
| RYQDFVKPGSANQDPIFTNNK | 2 | 2438.20 | 82232.84 | 38434.14 | 130.25 |
| WYAQAELMNAR | 0 | 1351.63 | 268352.24 | 36094.78 | 178.94 |
| YQDFVKPGSANQDPIFTNNK | 1 | 2282.10 | 520119.20 | 377366.16 | 209.43 |
| YQDFVKPGSANQDPIFTNNKLPDGNEPGYPGGIFDPFGWSK | 2 | 4513.13 | 24177.79 | 0.00 | 45.85 |
|  |  |  | 1630896.2 | 563022.5 |  |
